# Outcomes Linked to 3N2+1N1 Sampling by Surgery Type: A Commission on Cancer Lung Cancer Quality Metric

**DOI:** 10.1016/j.atssr.2025.09.012

**Published:** 2025-10-16

**Authors:** Alison S. Baskin, Haley I. Tupper, Varada Sarovar, Jessica Santhakumar, Kian C. Banks, Angela Sun, Rachel Wile, Katherine Barnes, Lori C. Sakoda, Jeffrey B. Velotta

**Affiliations:** 1 Division of General Surgery, Department of Surgery, University of California, San Francisco, San Francisco, California; 2 Division of General Surgery, Department of Surgery, University of California, Los Angeles, Los Angeles, California; 3 Division of Research, Kaiser Permanente Northern California, Pleasanton, California; 4 University of California, San Francisco School of Medicine, San Francisco, California; 5 Division of General Surgery, Department of Surgery, University of California, San Francisco East Bay, Oakland, California; 6 College of Letters and Sciences, University of California, Berkeley, Berkeley, California; 7 Department of Health Systems Science, Kaiser Permanente Bernard J. Tyson School of Medicine, Pasadena, California; 8 Department of Clinical Science, Kaiser Permanente Bernard J. Tyson School of Medicine, Pasadena, California; 9 Division of Thoracic Surgery, Department of Surgery, Kaiser Permanente Oakland, Oakland, California

## Abstract

**Background:**

In 2021, the Commission on Cancer implemented Standard 5.8 requiring lymph node sampling from ≥3 mediastinal and ≥1 hilar stations (3N2+1N1) during curative-intent lung cancer resections. Before Standard 5.8, sampling ≥10 lymph nodes was recommended. To date, the optimal nodal sampling strategy is still unknown, particularly for sublobar resections. We assessed 3N2+1N1 sampling patterns and potential associations with recurrence and mortality by resection type.

**Methods:**

In this multicenter retrospective study, we evaluated early-stage non-small cell lung cancer (NSCLC) patients who underwent lobectomy or sublobar resection (2009-2019). We calculated the proportion with 3N2+1N1 sampled. Using multivariable Cox regression, we assessed associations of 3N2+1N1 sampling with 1-year recurrence and 5-year overall mortality, stratified by lobectomy vs sublobar resection.

**Results:**

Among 2096 lobectomy patients, 43% had 3N2+1N1 sampling. In contrast, among 386 sublobar resection patients, 23% had 3N2+1N1. We found 3N2+1N1 sampling was not significantly associated with 1-year recurrence or 5-year mortality after lobectomy, but was associated with reduced 1-year recurrence (adjusted hazard ratio, 0.62; 95% CI, 0.39-0.98) after sublobar resection.

**Conclusions:**

A minority of lobectomy and sublobar resection patients had 3N2+1N1 sampling. Although 3N2+1N1 sampling was not associated with improvements across all outcomes, our findings suggest that Standard 5.8 may be a meaningful step toward improved quality of lymph node evaluations in some patients.

## Visual Abstract

**Figure:**
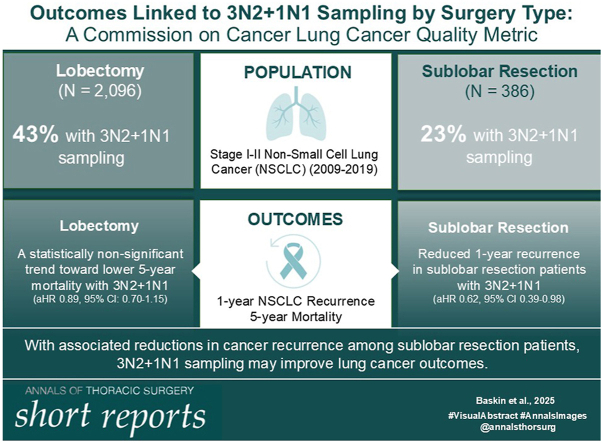

**In Short**

▪ Among sublobar resection patients, there was a particularly low proportion (23%) of 3N2+1N1 sampling.
▪ 3N2+1N1 sampling was linked to reduced 1-year recurrence in sublobar resections, suggesting a potential benefit from this sampling approach.

To address variation in the quality of intraoperative lymph node assessment, the American College of Surgeons Commission on Cancer introduced Standard 5.8 in 2021, requiring lymph node sampling from at least 3 distinct mediastinal (N2) nodal stations and 1 hilar (N1) station for all curative-intent lung cancer operations.^1^

Although limited lymph node evaluations have been associated with worse outcomes,^2^^,^^3^ conflicting data remain regarding the optimal extent of nodal sampling in sublobar resections.^4^^,^^5^ Most of these studies were conducted under previous guidelines focused on a minimum nodal count threshold and thus do not directly assess the 3N2+1N1 requirement. Moreover, emerging research has not stratified outcomes by extent of resection.^6^, ^7^, ^8^

Given Standard 5.8 was only recently implemented, understanding prior practice patterns is critical to inform ongoing and future quality improvement efforts. This study aimed to establish a preimplementation baseline for 3N2+1N1 sampling and assess early outcomes associated with this sampling approach. Specifically, we evaluated 3N2+1N1 sampling rates among non-small cell lung cancer (NSCLC) patients who underwent lobectomy or sublobar resection in the preimplementation period and analyzed cancer recurrence and mortality by resection type. We hypothesized that 3N2+1N1 sampling would be infrequent during the preimplementation period, especially for sublobar resections, but that it would be associated with improved outcomes.

## Patients and Methods

This data-only, multicenter, retrospective study was approved by the Kaiser Permanente Northern California (KPNC) Institutional Review Board (1610759), granted a waiver of informed consent, and is reported in accordance with the Strengthening the Reporting of Observational Studies in Epidemiology (STROBE) checklist.

### Study Population

We used an existing cohort of adult patients with clinical stage I or II NSCLC without prior lung cancer treatment who underwent curative-intent surgical resection between 2009 and 2019 at KPNC,^9^ an integrated health care delivery system serving 4.4 million patients. We excluded patients with pneumonectomy or bilobectomy, who had missing lymphovascular invasion data, an indeterminate clinical stage, or were diagnosed with pathologic stage IV disease within 90 days of surgery (Supplemental Figure).

### Data Collection

From KPNC administrative and clinical databases, we extracted diagnosis date, age, sex, race/ethnicity, neighborhood deprivation index, Charlson comorbidity index score, smoking history, history of non-NSCLC cancer in the past year, clinical and pathologic stage, histologic subtype, lymphovascular invasion, pathologic margin status, tumor laterality, and date and type of surgery (Supplemental Methods). Primary surgical resections were categorized as lobectomy or sublobar resection, including wedge resections and segmentectomies.

We evaluated rates of 3N2+1N1 sampling. The number and anatomical stations of sampled lymph nodes were extracted from surgical pathology reports. The primary outcomes were 1-year NSCLC recurrence and 5-year overall mortality.

### Statistical Analysis

**Figure 1:**
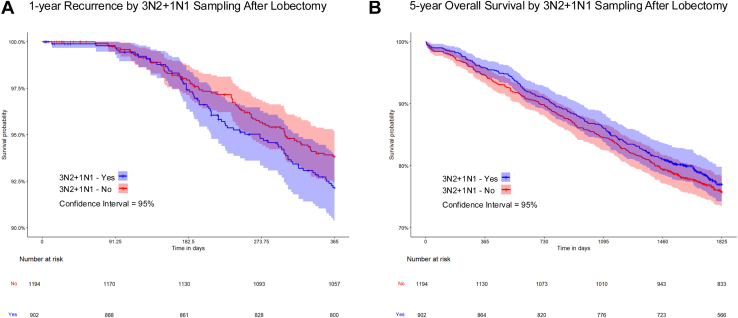
Kaplan-Meier curves of (A) 1-year recurrence and (B) 5-year mortality by 3N2+1N1 sampling among lobectomy patients. The shaded areas indicate the 95% CI.

**Figure 2:**
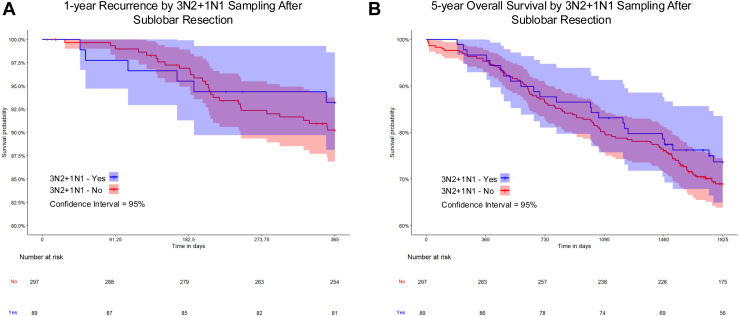
Kaplan-Meier curves of (A) 1-year recurrence and (B) 5-year mortality by 3N2+1N1 sampling among sublobar resection patients. The shaded areas indicate the 95% CI.

We used the Wilcoxon rank sum test, Pearson χ^2^ test, or Fisher exact test to compare characteristics between patients with 3N2+1N1 sampling, stratified into lobectomy and sublobar resection subgroups. Kaplan-Meier methods were used to estimate 1-year cancer recurrence and 5-year survival by whether 3N2+1N1 criteria were met (Figure 1, Figure 2).

We performed unadjusted and adjusted Cox regression analyses to evaluate the association of 3N2+1N1 with primary outcomes. We adjusted for covariates previously shown to influence prognosis: age, sex, race/ethnicity, neighborhood deprivation index, Charlson comorbidity index, smoking history, cancer in prior year, clinical stage, histologic subtype, lymphovascular invasion, and tumor laterality. All analyses were performed using R 4.3.1 software (R Project for Statistical Computing). Statistical tests were 2-sided with a *P* value <.05 considered as statistically significant.

## Results

Of the 2482 patients who met inclusion criteria, 2096 (84%) underwent lobectomy and 386 (16%) underwent sublobar resection. Among lobectomy patients, those from less-deprived neighborhoods, with clinical stage II disease, and lymphovascular invasion more commonly had 3N2+1N1 sampling (Table 1). Among sublobar resection patients, 3N2+1N1 sampling was more common in individuals with no prior smoking history and clinical stage II disease.

**Table 1:** Characteristics Associated With 3N2+1N1 Sampling

Characteristic	Lobectomy			Sublobar Resection		
	3N2+1N1		*P*^a^	3N2+1N1		*P*^a^
	Yes	No		Yes	No	
Patients	902 (43)	1194 (57)		89 (23)	297 (77)	
Age, y			.55			.10
18 to <65	267 (29.6)	333 (27.9)		25 (28.1)	63 (21.2)	
65 to <75	379 (42.0)	529 (44.3)		40 (44.9)	118 (39.7)	
75 to <85	256 (28.4)	332 (27.8)		24 (27.0)	116 (39.1)	
Sex			.36			.92
Female	552 (61.2)	707 (59.2)		53 (59.6)	175 (58.9)	
Male	350 (38.8)	487 (40.8)		36 (40.4)	122 (41.1)	
Race/ethnicity			0.55			0.56
White	566 (62.7)	784 (65.7)		54 (60.7)	199 (67.0)	
Asian	146 (16.2)	185 (15.5)		16 (18.0)	37 (12.5)	
Black	68 (7.5)	75 (6.3)		8 (9.0)	24 (8.1)	
Hispanic	62 (6.9)	69 (5.8)		8 (9.0)	21 (7.1)	
Other	60 (6.7)	81 (6.8)		3 (3.4)	16 (5.4)	
Neighborhood Deprivation Index, quartile			.002			.56
1 (least deprived)	264 (29.3)	267 (22.4)		27 (30.3)	69 (23.2)	
2	206 (22.8)	293 (24.5)		21 (23.6)	84 (28.3)	
3	225 (24.9)	303 (25.4)		23 (25.8)	79 (26.6)	
4 (most deprived)	207 (22.9)	331 (27.7)		18 (20.2)	65 (21.9)	
Charlson Comorbidity Index Score			.66			.42
0	154 (17.1)	218 (18.3)		7 (7.9)	25 (8.4)	
1-2	468 (51.9)	603 (50.5)		52 (58.4)	144 (48.5)	
3-4	200 (22.2)	253 (21.2)		19 (21.3)	80 (26.9)	
5+	80 (8.9)	120 (10.1)		11 (12.4)	48 (16.2)	
Smoking history			.83			.04
No	205 (22.7)	276 (23.1)		26 (29.2)	56 (18.9)	
Yes	697 (77.3)	918 (76.9)		63 (70.8)	241 (81.1)	
Other cancer in prior year			.61			.79
No	845 (93.7)	1,125 (94.2)		84 (94.4)	278 (93.6)	
Yes	57 (6.3)	69 (5.8)		5 (5.6)	19 (6.4)	
Clinical stage			< .001			.02
I	757 (83.9)	1,089 (91.2)		78 (87.6)	282 (94.9)	
II	145 (16.1)	105 (8.8)		11 (12.4)	15 (5.1)	
Histology			.85			.25
Adenocarcinoma	660 (73.2)	887 (74.3)		69 (77.5)	213 (71.7)	
Other	85 (9.4)	107 (9.0)		4 (4.5)	30 (10.1)	
Squamous cell	157 (17.4)	200 (16.8)		16 (18.0)	54 (18.2)	
Lymphovascular Invasion			< .001			.10
Negative	769 (85.3)	1,085 (90.9)		80 (89.9)	267 (89.9)	
Positive	133 (14.7)	109 (9.1)		9 (10.1)	30 (10.1)	
Pathologic margin status			.95			.21
Negative	882 (97.8)	1,168 (97.8)		89 (100.0)	289 (97.3)	
Positive	20 (2.2)	26 (2.2)		0 (0.0)	8 (2.7)	
Laterality			.20			.95
Left	365 (40.5)	450 (37.7)		44 (49.4)	148 (49.8)	
Right	537 (59.5)	744 (62.3)		45 (50.6)	149 (50.2)	

All data are presented as n (%).

a Pearson χ^2^; Fisher exact test.

Compared with sublobar resection patients, a greater proportion of lobectomy patients had 3N2+1N1 sampling (43% vs 23%; *P* < .001). Lobectomy patients had a higher median number of lymph nodes sampled (4.0 [interquartile range, 2-5] vs 2.5 [interquartile range, 1-4]; *P* < .001).

In adjusted analyses among lobectomy patients, 3N2+1N1 sampling was not associated with statistically significant differences in 1-year recurrence or 5-year mortality (Table 2). Among sublobar resection patients, 3N2+1N1 sampling was associated with reduced 1-year recurrence (adjusted hazard ratio, 0.62; 95% CI, 0.39-0.98), but no statistically significant difference in 5-year mortality.

**Table 2:** Outcomes Associated with 3N2+1N1 Sampling

Resection Type and Outcome	3N2+1N1	Events/Person-Days	HR (95% CI)	aHR^a^ (95% CI)
Lobectomy				
1-year recurrence	No	71/412,016	Reference	Reference
	Yes	69/312,633	1.28 (0.93-1.76)	1.10 (0.80-1.50)
5-year mortality	No	287/1,884,920	Reference	Reference
	Yes	203/1,427,973	0.93 (0.73-1.19)	0.89 (0.70-1.15)
Sublobar				
1-year recurrence	No	28/100,781	Reference	Reference
	Yes	6/30,963	0.70 (0.45-1.09)	0.62 (0.39-0.98)
5-year mortality	No	91/452,712	Reference	Reference
	Yes	23/138,277	0.83 (0.58-1.18)	1.00 (0.69-1.44)

aHR, adjusted hazard ratio; HR, hazard ratio.

a Adjusted for age, sex, race/ethnicity, neighborhood deprivation index, comorbidity score, smoking history, other cancer, clinical stage, histology, lymphovascular invasion, and tumor laterality.

## Comment

In this study of >2400 early-stage NSCLC patients who underwent surgery in the decade before the implementation of Commission on Cancer Standard 5.8, we evaluated baseline rates of 3N2+1N1 lymph node sampling. Consistent with published research, only 43% of lobectomies and 23% of sublobar resections met current 3N2+1N1 criteria.^6^, ^7^, ^8^ Unanimously, these findings align with emerging data on facility-level compliance with Standard 5.8 and underscore a potential opportunity for Standard 5.8 to reduce variation in the technical quality of surgical nodal evaluations across resection types.^10^

Our study helps address a knowledge gap by linking outcomes associated with 3N2+1N1 sampling to surgical resection type. Retrospective studies by Resio and colleagues^6^ and Heiden and colleagues^7^ found that 3N2+1N1 sampling is associated with reduced recurrence and improved survival in NSCLC, whereas Rocco and colleagues^8^ found no survival or recurrence benefit. None of these studies evaluated outcomes by surgical resection type.^6^, ^7^, ^8^ Importantly, we report that 3N2+1N1 sampling was associated with reduced 1-year NSCLC recurrence among sublobar resection patients; however, most sublobar resections did not meet standard criteria. This gap may be due to many reasons, including the greater technical complexity of performing thorough lymph node dissections during sublobar procedures, longer operative times, or prevailing beliefs that extensive sampling is unnecessary for small, peripheral, and/or ground-glass opacity or subsolid pulmonary nodules.

Our study has several limitations. As a retrospective analysis, it is subject to potential confounding. Despite adjustment for known variables, unknown confounders may still influence outcomes.

Second, our cohort included only early-stage NSCLC patients without neoadjuvant therapy, whereas Standard 5.8 applies more broadly. Nevertheless, NSCLC accounts for 80% to 85% of all lung cancer cases, and early-stage patients may derive the greatest benefit from comprehensive nodal evaluation given the risk of occult metastatic disease.

Third, we did not capture specific nodal stations or distinguish between sublobar resection types (ie, wedge resection and segmentectomy) given insufficient statistical power (Supplemental Table). Prospective studies with detailed nodal mapping and tumor-specific data are needed to better assess the impact of Standard 5.8.

In conclusion, this study underscores the importance of improving the quality of surgical lymph node evaluations for early-stage NSCLC. Low baseline 3N2+1N1 sampling rates suggest practice changes may be necessary to comply with new requirements. Although 3N2+1N1 sampling was not associated with improvements across all outcomes, benefits may be underestimated, given limited use of modern adjuvant therapies in our cohort. Although the nodal staging approach remains uncertain, our findings indicate that Standard 5.8 may be a meaningful step toward higher quality lung cancer surgery.
