# Supplementary material for: Outcomes Linked to 3N2+1N1 Sampling by Surgery Type: A Commission on Cancer Lung Cancer Quality Metric
Source: Ann Thorac Surg Short Rep. 2025 Oct 16;4(1):207–12. doi: 10.1016/j.atssr.2025.09.012 (PMC13100740; doi:10.1016/j.atssr.2025.09.012)
Supplement: Supplementary Table 1 [file mmc1.docx]

**Supplemental Table 1. Lymph Node Sampling Among Sublobar Resection Patients**

| **Lymph Node Sampling** | **All Sublobar**  N = 386 | **Wedge Resection,**  N = 323 (84%) | **Segmentectomy,**  N = 63 (16%) |
| --- | --- | --- | --- |
| 3N2+1N1 Sampled |  |  |  |
| No | 297 (77%) | 264 (82%) | 33 (52%) |
| Yes | 89 (23%) | 59 (18%) | 30 (48%) |
| 3N2 Nodes Sampled |  |  |  |
| No | 228 (59%) | 200 (62%) | 28 (44%) |
| Yes | 158 (41%) | 123 (38%) | 35 (56%) |
| 1N1 Nodes Sampled |  |  |  |
| No | 250 (65%) | 242 (75%) | 8 (13%) |
| Yes | 136 (35%) | 81 (25%) | 55 (87%) |
