# Supplementary material for: Outcomes Linked to 3N2+1N1 Sampling by Surgery Type: A Commission on Cancer Lung Cancer Quality Metric
Source: Ann Thorac Surg Short Rep. 2025 Oct 16;4(1):207–12. doi: 10.1016/j.atssr.2025.09.012 (PMC13100740; doi:10.1016/j.atssr.2025.09.012)
Supplement: Supplementary Material [file mmc2.docx]

**Supplementary Methods: Variable and Outcome Definitions**

| The diagnosis date was defined as date of the diagnostic computed tomography (CT) with findings suspicious for lung cancer. Age was categorized into three groups: 18 to <65 years, 65 to <75 years, and 75 to <85 years. Self-reported race and Hispanic ethnicity were reported together; race was omitted if the patient identified as Hispanic. Neighborhood deprivation index (NDI) is a validated census-based socioeconomic status measure derived from 13 different measures of wealth and income, education level, occupation, and housing conditions obtained from the Census Bureau’s 5-year American Community Survey. NDI scores range from -3.6 to 2.8, with higher values indicating greater neighborhood deprivation. In this study, NDI was divided into quartiles, with quartile 1 representing the least deprived neighborhood and quartile 4 representing the most deprived neighborhood. Charlson comorbidity index (CCI), which ranges from 0 to 4, was categorized as 0 (none), 1 to 2 (mild), 3 to 4 (moderate), and ≥5 (severe). Patients with a non-NSCLC diagnosis recorded in the electronic health record within one year prior to surgery were classified as having “other cancer in prior year.” The AJCC TNM cancer staging system (version 6, 7, or 8) corresponding to the year of NSCLC diagnosis was used. Histologic subtypes were classified as adenocarcinoma, squamous cell carcinoma, or “other”.  The date of recurrence was defined as the imaging date triggering a treatment change or the date of biopsy, if no imaging was available. Mortality data were captured from Kaiser Permanente Northern California (KPNC) databases, which included the latest mortality data from the state of California and National Death Index files. Consistent with CoC Standard 5.8 guidelines, lymph nodes sampled during same day mediastinoscopy were included in the intraoperative lymph node totals if documented in the primary lung resection’s pathology report.  Patients were followed from the date of surgery until the outcome of interest, death (when examining non-mortality outcomes), health plan disenrollment, or study end (December 31, 2023), whichever occurred first. |
| --- |
